# Medical advertising in social networks: awareness and medical school education

**DOI:** 10.1590/0100-6991e-20223386_en

**Published:** 2022-10-26

**Authors:** KÁTIA SHEYLLA MALTA PURIM, FABIANA ANTUNES DE ANDRADE, FERNANDA LEHMKUHL, AFRÂNIO BENEDITO SILVA BERNARDES

**Affiliations:** 1 - Universidade Positivo, Escola de Ciências da Saúde - Curso de Medicina - Curitiba - PR - Brasil

**Keywords:** Marketing of Health Services, Education, Medical, Students, Medical, Computer Security, Biomedical Technology, Publicidade, Educação Médica, Estudantes de Medicina, Proteção de Dados, Tecnologias em Saúde

## Abstract

**Objective:**

this study analyzed medicine students’ knowledge regarding medical advertising on social media.

**Method:**

this is a cross-sectional study carried out between January and May 2022 with 179 medical students from public and private institutions from Curitiba - PR, using a structured questionnaire with nine problem situations on medical advertising. It was established as “sufficient” knowledge ≥70% of the problem-situations based on current professional codes and resolutions.

**Results:**

five questions had the highest percentage of correct answers resulting from the acquisition of knowledge from different sources. Most students did not learn about medical marketing in their undergraduate course (84.9%), having already shared patients’ pictures on social media (89.9%), and fell the lack of discussions about medical advertising (96.6%).

**Conclusion:**

there is a need to direct undergraduate education towards the ethical use of advertising in order to better prepare them for professional practice.

## RESUMO

**Objetivo:**

este estudo analisou o conhecimento dos estudantes de medicina sobre publicidade médica nas redes sociais.

**Método:**

Trata-se de estudo transversal realizado entre janeiro e maio de 2022 com 179 acadêmicos de medicina de instituição pública e privada em Curitiba - PR, por meio de questionário estruturado com nove situações-problemas sobre publicidade médica. Foi estabelecido como conhecimento “suficiente” o acerto ≥70% das situações-problemas com base nos códigos e resoluções profissionais vigentes.

**Resultados:**

cinco questões tiveram maior percentual de acerto decorrente da aquisição de conhecimento de diferentes fontes. A maioria dos estudantes não aprendeu sobre publicidade médica na graduação (84,9%), já divulgou imagens de pacientes pelas redes sociais (89,9%), e sente falta de mais discussões sobre publicidade (96,6%).

**Conclusão:**

há necessidade de direcionar o ensino da graduação para o uso ético da publicidade visando melhor preparo para o exercício profissional.

## INTRODUCTION

Appropriate advertising and marketing strategies can educate the population, provide updated and accurate information to the patient, and give visibility to the physician, as more people look for referrals on the internet. Digital medical marketing is an emerging issue, which can be understood as “a set of actions and strategies that aim at adding value to medical practice through the identification of opportunities and markets and the desires and needs of patients”^1^.

The Federal Council of Medicine (CFM), through the Code of Medical Ethics (CEM)^2^ and Resolutions 1974/2011, 2126, and 2133/2015, regulates medical advertising for all advertising vehicles^3^. Through the Regional Councils of Medicine, it promotes updated and continuing education^4^ ^,^ ^5^ in various subjects for students and professionals, aiming at good medical practice and at building a solid career.

The General Data Protection Law (LGPD - Brazilian version of GDPR)^5^ ^,^ ^6^ considers health related data (diseases, disabilities, medical reports, medical records, biometric data, test results, among others) to be sensitive^6^, and use of this data can only be done with the authorization of its bearer and in compliance with legal provisions. On their turn, physicians and medical students are subject to professional confidentiality^2,7 10^.

With the popularization of social networks and the advent of the pandemic, there was an increase in distance learning and work, and digital communication intensified in all areas. Added to these is the growing number of vacancies in Medical Schools and the consequent increase in the supply of professionals to the market^9^ ^,^ ^11^ publicizing medical matters and services. The ability to communicate ethically, privately or in public, through verbal and non-verbal language, is one of the bases of professional practice provided for in the National Curriculum Guidelines (DCN) for medical courses^12^. However, most of the time, medical advertising is relegated to the hidden curriculum and is poorly understood by students.

The Medical Student Code of Ethics (CEEM)^7^ guides attitudes, practices, and moral and ethical principles inside and outside the classroom and establishes the rights and duties of undergraduates towards their peers, teachers, and patients. This study thus aims at analyzing the knowledge of medical students about medical advertising on social networks.

## METHODS

We carried out a cross-sectional, descriptive study between January and May 2022, through the Google Forms platform, available in WhatsApp groups of medical students in Curitiba-PR. The project was previously approved by the Ethics in Research Committee of the Universidade Positivo (CAAE 10718919.9.0000.0093).

The sample was non-probabilistic, having as inclusion criteria medical students over 18 years of age who agreed to participate by means of an informed consent form (ICF) and who returned the completed questionnaire. We excluded questionnaires with less than 80% of filled items, medical students from other countries, and exchange students.

We collected data through a structured questionnaire applied anonymously and online, containing the ICF on its homepage, and considering sociodemographic variables (age, sex, color/race/ethnicity, marital status, educational origin, private institution or public), questions about academic training, and knowledge about medical advertising. They were asked if they knew the Medical Publicity Manual (MPM - Resolution CFM 1974/2011), the Code of Ethics for Medical Students (CEEM), and if they had already attended the Bioethics course or equivalent, as well as sources of information about medical publicity.

**Chart 1:**
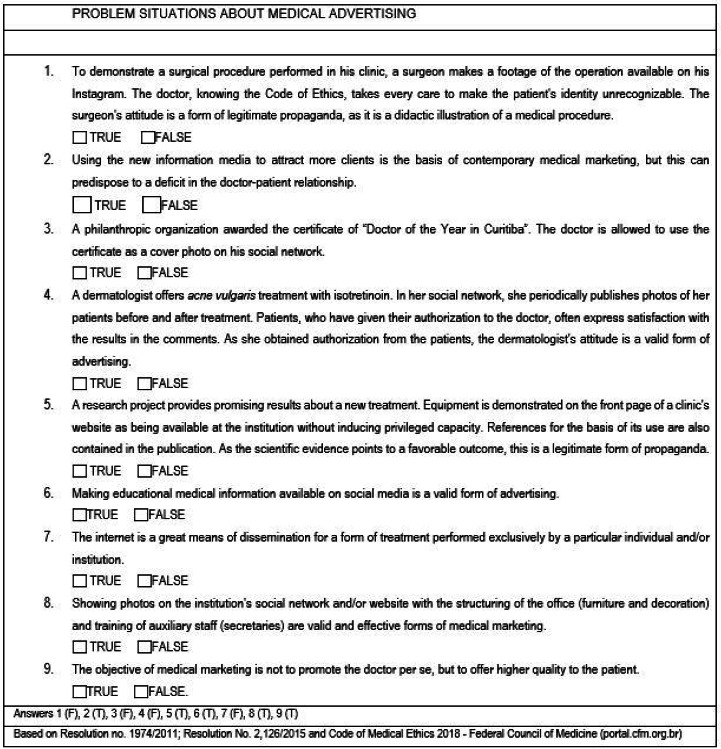
Problem-situations on medical advertising in view of the resolutions and professional codes in force in Brazil.

We inserted problem cases (Chart 1) describing everyday situations related to professional marketing for the student to answer “true” or “false” regarding the current ethical principles that govern medicine (Resolution 1974/2011; Resolution 2015), with the degree of knowledge classified as “insufficient” (<70% of correct answers) and “sufficient” (≥70% of correct answers).

We tabulated the data in Microsoft Excel^®^ spreadsheets. We performed descriptive and inferential statistics using tests based on the Chi-Square distribution and Fisher’s exact test, considering p<0.05 as significant. All analyzes were performed using the SPSS 17.0 program.

## RESULTS

The sample comprised 179 students, with a predominance (Table 1) of women (64.8%), young individuals (66.5%), white (89.9%), single (94.4%), coming from a private institution (52.5%), and who had already attended the Bioethics course or equivalent (96.1%). The Medical Publicity Manual was known by 54.2% of the students and the CEEM, by 50% (Table 2). 

**Table 1:** Characteristics of the sample of medical students (n=179).

Features	n	%
Sex		
Female	116	(64.8)
Male	63	(35.2)
Marital status		
Married	8	(4.5)
Other	2	(1.1)
Single	169	(94.4)
Skin color		
Yellow	9	(5.0)
White	161	(89.9)
Black	1	(,6)
Brown	8	(4.5)
Age group		
Less than 20 years	24	(13.4)
20-24 years	119	(66.5)
Features	n	%
25-29 years	30	(16.8)
30-34 years	4	(2.2)
35-39 years	1	(0.6)
40 years or more	1	(0.6)
Institution of origin		
Private	94	(52.5)
Public	85	(47.5)

**Table 2:** Training and professionalization of medical students (n=179).

Data on training and professionalization	n	%
Did you take the bioethics course or equivalent during graduation?		
No	7	(3.9)
Yes	172	(96.1)
Do you know the Medical Advertising Manual of the Federal Council of Medicine?		
No	82	(45.8)
Yes	97	(54.2)
Do you know the medical student ethics manual?		
No	89	(50.0)
Yes	90	(50.0)
Did you learn about medical advertising as an undergraduate?		
No	152	(84.9)
Yes	27	(15.1)
How/where did you learn about medical advertising? Professors?		
No	154	(86.0)
Yes	25	(14.0)
Data on training and professionalization	n	%
Did you learn through conferences?		
No	139	(77.7)
Yes	40	(22.3)
Did you learn through academic leagues?		
No	121	(67.6)
Yes	58	(32.4)
Did you learn through extracurricular events?		
No	97	(54.2)
Yes	82	(45.8)
Did you learn through contact with other doctors?		
No	164	(91.6)
Yes	15	(8.4)
Did you learn through the Internet?		
No	92	(51.4)
Yes	87	(48.6)
Do you consider medical advertising training important for professional practice?		
No	117	(65.4)
Yes	62	(34.6)
Do you miss more discussions about professional advertising in medical education?		
No	6	(3.4)
Yes	173	(96.6)
Have you shared images of patients on social media?		
No	18	(10.1)
Yes	161	(89.9)

The percentage of students who did not learn about medical advertising in the undergraduate course was 84.9%, and who did not consider this training important for their professional practice, 65.4%. Most had already shared images of patients on social networks (89.9%), felt a lack of more discussions about medical advertising (96.6%), and sought to acquire this knowledge from different sources (Table 2). 

When comparing medical students who attended the Bioethics Discipline or not, there was a significant difference in the influence of graduation (p=0.031) and professors (p=0.007) as a source of learning.

As for the dissemination of images of patients through social networks, we found no statistical difference regarding the knowledge arising from the Medical Publicity Manual (p=0.702), CEEM (p=0.498), gender (p=0470), private or public institution (p=0.311), learning through the internet (p=0120), or having attended a Bioethics course (p=0.414).

**Figure 1:**
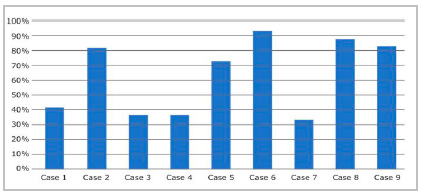
Percentage of students’ correct answers regarding questions about medical advertising.

Regarding questions about medical advertising, the lowest percentages of correct answers occurred in the situations listed in case 1 (41.3=3%), 3, 4 (both with 37.4%), and 7 (33.5%) (Figure 1).

**Figura 2:**
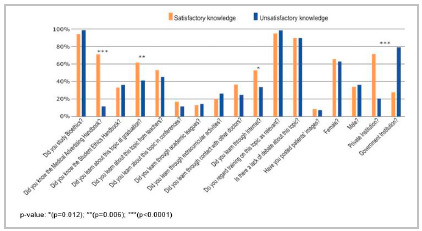
Student’s knowledge about medical advertising considering variables related to training/professionalization.

Knowledge about medical advertising was unsatisfactory among those who reported knowing the Medical Advertising Manual (p<0.0001), who learned it during graduation (p=0.006), through the internet (p=0.012), and who had come from a private institution (p<0.0001) (Figure 2).

## DISCUSSION

The profile of students showed a predominance of female and young individuals, consistent with the literature^8^ ^,^ ^13^, and points out that, despite the curricular guidelines encouraging the teaching of communication skills^12^, there is an educational gap regarding advertising and professional marketing during medical graduation^9^.

In the present study, most undergraduates attended the Bioethics course and knew the Medical Publicity Manual, but only half knew the CEEM. In addition, knowledge on the subject was unsatisfactory among those who were familiar with the Medical Advertising Manual. Approaching ethics in a creative, reflective, and contextualized way throughout the undergraduate course can help improve professionalism^8^. The practices and attitudes of undergraduates^7^, inside and outside the virtual environment, must be aligned with the rules of moral conduct, current legislation, and the expected behavior of a future doctor^3^ ^,^ ^13^ ^-^ ^18^.

When delimiting where the general knowledge of the medical student about medical advertising comes from, in this sample we observed that the formal curriculum, the professors, and the interaction with other doctors had indications of lower participation and that the students claimed to miss the teaching advertising in their training. On the contrary, they stated that they do not consider this training important for professional practice. However, with the technological dynamics, cyberculture in health, changes in the doctor-patient relationship, and the expansion of telemedicine, this skill complements the construction of the contemporary professional profile^9^ ^,^ ^15^.

In this sample, remote teaching and social distancing due to the SARS-CoV-2 pandemic may have influenced the relationship with professors and interaction with doctors, as well as the perception of advertising. Other possible explanations would be that, although digital natives, students have not critically reflected on how mass communication vehicles can impact the population’s health choices^16^ ^,^ ^17^.

The distribution of content on the internet can influence the public perception of the profession and one’s own personal image, and according to a survey carried out with medical students, the use of social networks can have positive and negative implications^13^.

The dissemination of images of patients on social networks by undergraduates, detected in the present study, is worrying and probably reflects a lack of knowledge of the legislation, codes, and norms and their consequences^1^ ^,^ ^8^ ^,^ ^18^. On the other hand, it may have been motivated by impulsiveness and the frequent placement in virtual social spaces and lay media of concrete cases, to increase audience and gain followers. Thus, students should be educated about medical confidentiality^2^ and legislation5, to ensure the reliability and integrity of their actions, as well as to reinforce safety behavior and privacy policies to prevent, monitor, and mitigate risks^6^.

In the present study, the postings of patients’ images did not show statistical differences in relation to gender, educational institution, attendance of Bioethics, learning through the internet, and knowing the CEEM or the Medical Publicity Manual, and may result from socio-behavioral characteristics of each student. This finding should be further investigated and serves as an alert to clarify medical ethics for students, due to the risk of breach of confidentiality and sanctions in the moral, legal, and administrative scope^1^ ^,^ ^17^.

Students showed a higher percentage of ignorance of current regulations on medical advertising in problem cases 1, 3, 4, and 7 (Chart 1, Figure 1), medical ethics in social media (websites, blogs, Facebook, Twitter, Instagram, YouTube, WhatsApp, and the like) being addressed by the CFM Resolution No. 2,126/2015^14^. The idea is not to censor or restrict the professional’s activity, but to establish parameters for an ethical and healthy practice, avoiding abuse and unnecessary exposure, respecting the human being and the educational purpose of medical advertising (Article 111 of the CEM)^2^.

The trend of the 21^st^ century is the provision of medical services in a hybrid way, in person and through Telemedicine (Resolution CFM 2314 / 2022, article 3), whose regulations also determine that “the data and images of the patients contained in the medical records must be preserved, complying with the legal and CFM rules pertaining to the safekeeping, handling, integrity, veracity, confidentiality, privacy, irrefutability, and guarantee of professional secrecy of information”^15^.

The limitations of the present study are its design, possible self-selection, and response biases, as the questionnaire was distributed to medical students’ social media and self-completed. Although subject to criticism, its findings reveal the importance of teaching medical advertising and provoke the reflection that the training of digital bioethics and telepropaedeutics is urgent for the future exercise of the medical profession^2^ ^,^ ^7^.

The Medical Student Medical Ethics Code and the rules of professional advertising can be included in the Bioethics Discipline or equivalent, due to its magnitude, as well as implementing curricular activities to train and evaluate adequate communication and digital marketing strategies, using active methodologies, and extend its teaching to internships and medical residency programs. However, it is up to the medical students to be the protagonists of their learning^13^ and their professionalism^7^ ^,^ ^8^ ^,^ ^18^, and to continually improve their knowledge to apply the best of scientific progress, technologies, and training for the benefit of their patients and society.
